# Tamoxifen activity against *Plasmodium* in vitro and in mice

**DOI:** 10.1186/s12936-019-3012-7

**Published:** 2019-11-27

**Authors:** Ada Weinstock, Julio Gallego-Delgado, Cláudia Gomes, Julian Sherman, Cyrus Nikain, Sandra Gonzalez, Edward Fisher, Ana Rodriguez

**Affiliations:** 1 Departments of Medicine (Cardiology) and Cell Biology, and the Marc and Ruti Bell Program in Vascular Biology, New York University School of Medicine, New York, NY 10016 USA; 2 Department of Biological Sciences, Lehman College, City University of New York, Bronx, New York, NY 10468 USA; 3 Ph.D. Program in Biology, The Graduate Center, The City University of New York, New York, NY 10016 USA; 4 Department of Microbiology, New York University School of Medicine, New York, NY 10016 USA

**Keywords:** Tamoxifen, 4-Hydroxytamoxifen, Cerebral malaria, *Plasmodium berghei*, *Plasmodium falciparum*, Cre/LoxP

## Abstract

**Background:**

Tamoxifen is an oestrogen receptor modulator that is widely used for the treatment of early stage breast cancer and reduction of recurrences. Tamoxifen is also used as a powerful research tool for controlling gene expression in the context of the Cre/loxP site-specific recombination system in conditional mutant mice.

**Methods:**

To determine whether the administration of tamoxifen affects *Plasmodium* growth and/or disease outcome in malaria, in vitro studies assessing the effect of tamoxifen and its active metabolite 4-hydroxytamoxifen on *Plasmodium falciparum* blood stages were performed. Tamoxifen effects were also evaluated in vivo treating C57/B6 mice infected with *Plasmodium berghei* (ANKA strain), which is the standard animal model for the study of cerebral malaria.

**Results:**

Tamoxifen and its active metabolite, 4-hydroxytamoxifen, show activity in vitro against *P. falciparum* (16.7 to 5.8 µM IC50, respectively). This activity was also confirmed in tamoxifen-treated mice infected with *P. berghei*, which show lower levels of parasitaemia and do not develop signs of cerebral malaria, compared to control mice. Mice treated with tamoxifen for 1 week and left untreated for an additional week before infection showed similar parasitaemia levels and signs of cerebral malaria as control untreated mice.

**Conclusions:**

Tamoxifen and its active metabolite, 4-hydroxytamoxifen, have significant activity against the human parasite *P. falciparum* in vitro and the rodent parasite *P. berghei* in vivo. This activity may be useful for prevention of malaria in patients taking this drug chronically, but also represents a major problem for scientists using the conditional mutagenic Cre/LoxP system in the setting of rodent malaria. Allowing mice to clear tamoxifen before starting a *Plasmodium* infection allows the use the Cre/LoxP conditional mutagenic system to investigate gene function in specific tissues.

## Background

Tamoxifen is the most used selective oestrogen-receptor modulator to prevent and treat oestrogen receptor positive breast cancer. More than 40 years after tamoxifen discovery, it still remains as one of the most effective therapies for cancer treatment [1]. Due to its low cost and safety profile, its use is now generalized worldwide [2].

Tamoxifen is also well known by the scientific community, not only for its outstanding anticancer properties, but also for its role as exogenous ligand for the inducible Cre/loxP site-specific recombination system. The Cre/loxP site-specific recombination system is a powerful and extensively used tool for controlling gene expression. The Cre/LoxP system allows researchers to tightly control gene expression in a tissue and time specific manner. These conditional mutagenic systems consist of the gene for Cre-recombinase fused to mutated hormone-binding domains of the oestrogen receptor that can be activated by administration of tamoxifen to the animal [3]. The power and versatility of this resource has become an essential tool for gene targeting experiments in almost every medical research field. Surprisingly, published studies using this conditional mutagenic technology in the setting of experimental malaria, where rodent infections are the most common model used for research in this disease, were not found.

The last World Health Organization (WHO) malaria report confirms an estimated 219 million new cases of malaria and more than 400,000 deaths, approximately the same number observed during the previous 2 years [4]. The stagnation in the reduction of cases and deaths, keeps malaria as one of the top 5 causes of death in children worldwide, claiming a child’s life every 2 min [5]. The combination of experimental models of malaria and Cre/loxP conditional mutagenic system would be a powerful strategy to further understand the mechanisms regulating susceptibility to malaria. However, this system requires the use of tamoxifen to induce Cre/loxP recombination in mice.

Tamoxifen has shown additional activities other than anticancer including antibacterial, antiviral and even antiparasitic (anti-echinococcus and anti-leishmania) effects [1]. Moreover, tamoxifen interferes in vitro with the glucosylceramidesynthase and sphingomyelin synthase levels of *Plasmodium falciparum* affecting the bioactive sphingolipid pathway [6]. Although it has been reported that tamoxifen has no effect over *P. falciparum* growth at concentrations lower than 10 µM [7], there are no studies about the effect of this drug at higher concentrations in vitro or on other *Plasmodium* spp. in vivo.

In this work, the activity of tamoxifen and its active metabolite 4-hydroxytamoxifen in vitro against *P. falciparum* was evaluated. The impact of tamoxifen in *Plasmodium berghei* infections in mice was also analysed, including parasitaemia levels and the development of neurological signs in the experimental cerebral malaria mouse model. Finally, an alternative protocol that allows the use of Cre/loxP conditional mutagenic system for the study of *Plasmodium* infections and development of cerebral malaria in mouse models is provided.

## Methods

This study strictly followed the recommendations in the Guide for the Care and Use of Laboratory Animals of the National Institutes of Health. The protocol was reviewed and approved by the Institutional Animal Care and Use Committee of New York University School of Medicine, which is fully accredited by the Association for Assessment and Accreditation Of Laboratory Animal Care International (AAALAC).

### Determination of IC50 against *Plasmodium falciparum* in vitro

Using 96-well plates, 100 µl of *P. falciparum* growth medium (RPMI 1640, 25 mM HEPES, 0.1 mg/ml gentamicin, 0.05 mg/l hypoxanthine [pH 6.75]), supplemented with 0.25% sodium bicarbonate and 0.5% Albumax II (Invitrogen), were added to each well. Compounds from a DMSO stock at 10 mM were then serially diluted in triplicate. DMSO at the highest concentration used (0.5%) was tested in parallel, showing no differences from the control samples.

*Plasmodium falciparum* infected erythrocytes (RBCs) were incubated at 0.25% parasitaemia and 5% haematocrit and treated with increasing doses of tamoxifen, its bioactive metabolite 4-hydroxytamoxifen, and chloroquine as positive control (IC50 < 0.78 µM), for 96 h in a gas chamber containing 90% nitrogen, 5% carbon dioxide and 5% oxygen. After incubation, the parasite was frozen for 24 h at − 80 °C, then thawed for 4 h and transferred to a new 96-well plate. 0.01% SYBR Green I nucleic acid staining dye (Molecular Probes) mixed with lysis buffer (20 mM Tris at pH 7.5, 5 mM EDTA, 0.008% saponin, and 0.08% Triton X-100) was added to each well and incubated at room temperature on a shaker for 1 h. Fluorescence was measured using excitation and emission wavelengths of 485 and 530 nm, respectively.

### Activity of tamoxifen against *P. falciparum* blood stage in vitro

*Plasmodium falciparum* cultures were synchronized using sorbitol 5%. Synchronized cultures at ring/trophozoite or schizont/ring stages were incubated with 4-hydroxytamoxifen at 25 μM or DMSO 0.5% as a control for 24 or 18 h and parasite densities were quantified.

### Experimental *P. berghei* infections

Both males and females 5 weeks old C57/B6 mice were purchased from Taconic Farms Inc. Animals were infected by i.p. inoculation of 200 µl of sterile PBS containing different concentrations of *P. berghei* (ANKA strain) infected RBCs (1 × 10^6^, 5 × 10^6^ or 20 × 10^6^). Parasitaemia was determined in Giemsa-stained thin blood smears by microscopy. Determination and severity of experimental cerebral malaria was scored as previously described [8] based on appearance (Normal = 0; Coat ruffled = 1; Coat staring/panting = 2) and behaviour (Normal = 0; Hunched = 1; Partial paralysis = 3; Convulsions = 4). Tamoxifen was administered in the diet (Envigo) at 40 mg/kg per day.

### Statistical analyses

Significance for intra-erythrocytic *P. falciparum* growth was calculated using 2-way ANOVA. Significance for parasitaemia was calculated for each day individually, using unpaired t test for experiments with 2 groups and 1-way ANOVA for experiments with more than 2 groups. The area under the curve was calculated for disease score and statistical analysis was performed using an unpaired t test. Survival curves were compared using Log-rank (Mantel–Cox) test. p ≤ 0.05 was considered significant. Data was analysed using GraphPad Prism 7.05.

## Results

### Tamoxifen inhibits *P. falciparum* growth in vitro

To test whether tamoxifen has any direct effects on *P. falciparum* growth, an in vitro growth assay was performed. The parasite was treated with increasing doses of tamoxifen, or its bioactive metabolite 4-hydroxytamoxifen [9]. Parasite viability was measured after 96 h.

**Fig. 1:**
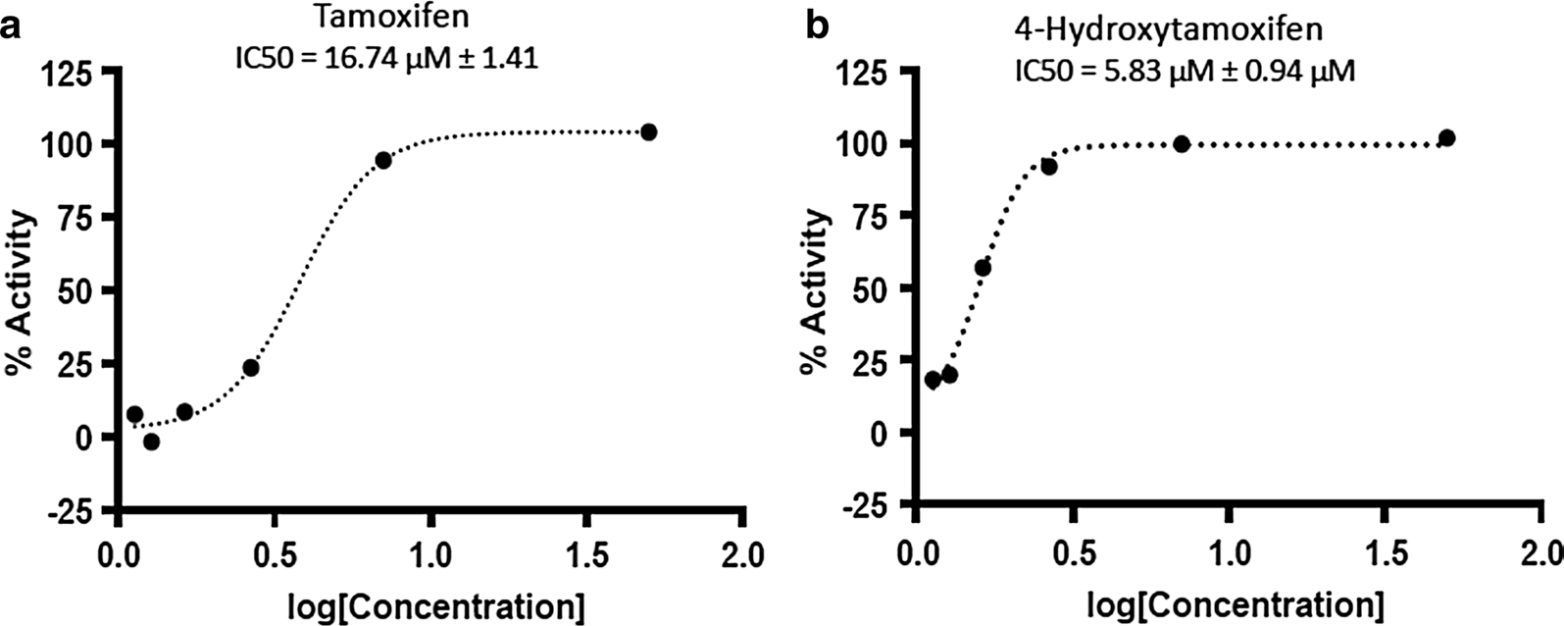
Determination of Tamoxifen antiplasmodial activity in vitro. *Plasmodium falciparum* cultures were incubated with increasing doses of tamoxifen (**a**) or 4-hydroxytamoxifen (**b**) for 96 h. Parasite viability was measured using SYBR Green fluorescence and the half-maximal inhibitory concentration (IC50) was calculated for each drug. Results show the average and standard deviation of triplicated determinations for each concentration

These results show that tamoxifen and 4-hydroxytamoxifen have IC50s against *P. falciparum* of 16.7 µM and 5.8 µM, respectively (Fig. 1). These results indicate that the tamoxifen bioactive metabolite is more effective than the original compound, predicting a significant in vivo anti-Plasmodium activity.

**Fig. 2:**
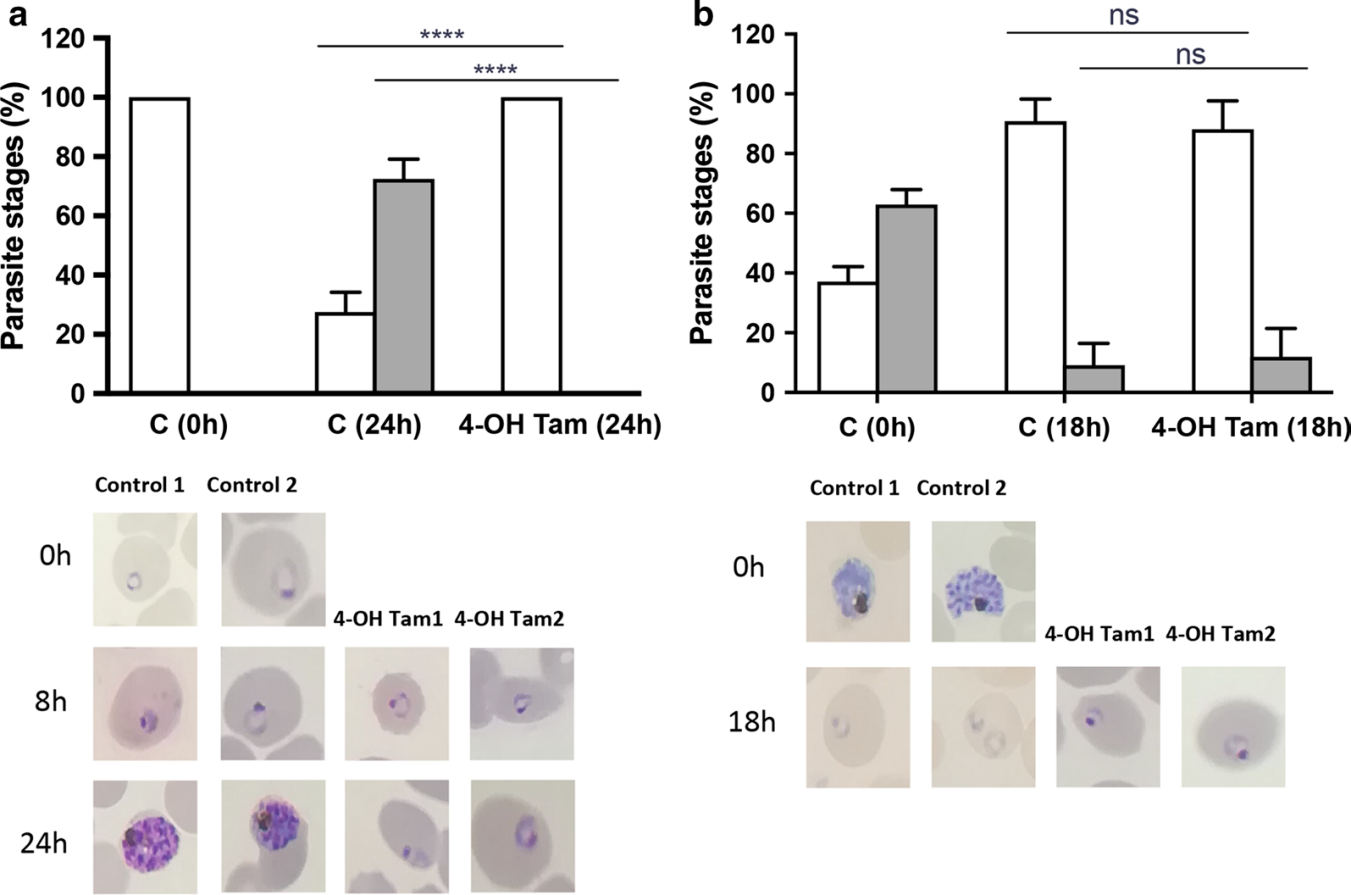
Tamoxifen active metabolite, 4-hydroxytamoxifen, inhibits intra-erythrocytic *P. falciparum* growth. *Plasmodium falciparum* cultures synchronized at ring/trophozoite (**a**) or schizont (**b**) stages at the beginning of the experiment were incubated with vehicle (DMSO 0.5%) as a control (**c**), or 4-hydroxytamoxifen (4-OH Tam) at 25 μM for 24 h (**a**) or 18 h (**b**). Quantification results for parasite stages at 24 h (**a**) or 18 h (**b**) after addition of tamoxifen show average and standard deviation of quadruplicated determinations for rings/trophozoites (white bars), and schizonts (gray bars). Statistical significance was calculated using 2-way ANOVA (****p < 0.0001), ns (not significant). The panels show representative images of parasites at the indicated time points

To characterize in more detail the effect of 4-hydroxytamoxifen on *P. falciparum*-infected RBCs, this compound was added to in vitro-synchronized cultures of the parasite and followed their development. When 4-hydroxytamoxifen was added to cultures of ring/trophozoite stages for 24 h, an almost complete arrest of parasite growth was observed (Fig. 2a), indicating that 4-hydroxytamoxifen affects early intra-erythrocytic parasite development. The addition of 4-hydroxytamoxifen to cultures in the schizont stage for 18 h did not affect the progression from schizonts into rings (Fig. 2b), indicating that merozoite release and reinvasion of new RBCs was not affected.

### Tamoxifen inhibits *P. berghei* growth and the development of cerebral malaria in mice

**Fig. 3:**
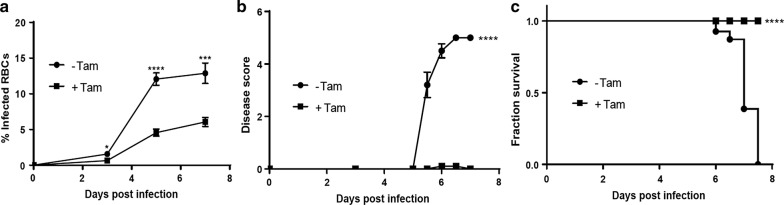
Tamoxifen has antiplasmodial activity in vivo and inhibits experimental cerebral malaria in mice. CB57/B6 mice were treated with tamoxifen 40 mg/kg per day 1 week prior infection with *P. berghei* ANKA and during the course of the infection (squares) versus untreated control group (circles). Animals treated with tamoxifen exhibited lower parasitaemia levels (**a**), lower neurological signs of cerebral malaria (**b**) and a 100% survival ratio after 7 days post-infection (**c**) compared with control group. Statistical significance was calculated using an unpaired t test (**a**, **b**) or Log-rank (Mantel–Cox) test (**c**) (*p < 0.05; ***p < 0.01; ****p < 0.0001)

Since tamoxifen and its bioactive form impair parasite growth in vitro, the tamoxifen effect on infected mice was examined. For this purpose, C57/B6 mice were treated with tamoxifen for 1 week prior to infection with 10^6^ *P. berghei* infected RBCs. Tamoxifen treatment continued throughout the course of infection. At day 3 post infection, parasitaemia was significantly higher in the control group (1.56% ± 0.39) compared with the tamoxifen treated mice (0.64% ± 0.1). The differences in parasitaemia increased over time with a 2.7-fold difference between control and treated mice at day 5 post infection that continued at day 7 (Fig. 3a).

Mice were closely monitored for the development of neurological signs of cerebral malaria. Interestingly, mice treated with tamoxifen did not develop cerebral malaria. At day 6 post infection 100% of the control mice (− Tam), compared to 10% of tamoxifen-treated mice (+ Tam), presented typical neurological signs associated with cerebral malaria (Fig. 3b). Additionally, the severity of the disease was much greater in the control mice, as indicated by the disease score (Fig. 3b). Accordingly, the control group presented significantly higher mortality. At day 7.5 post infection, all of the tamoxifen treated mice had survived while all of the control animals had died (Fig. 3c). The tamoxifen treated group was monitored for additional 2 weeks, in which none of the mice showed signs of cerebral malaria. Taken together, these data demonstrate that tamoxifen treatment inhibits parasite growth and prevents the development of cerebral malaria.

### Alternative strategies to study cerebral malaria in genetic mouse models that require tamoxifen

Finding that tamoxifen inhibits the development of cerebral malaria in mice indicates that the use of the Cre/LoxP recombination system in mice cannot be used for the study of cerebral malaria. To overcome this limitation, an attempt was made to find the conditions to induce cerebral malaria in mice treated with tamoxifen to induce Cre/LoxP recombination.

**Fig. 4:**
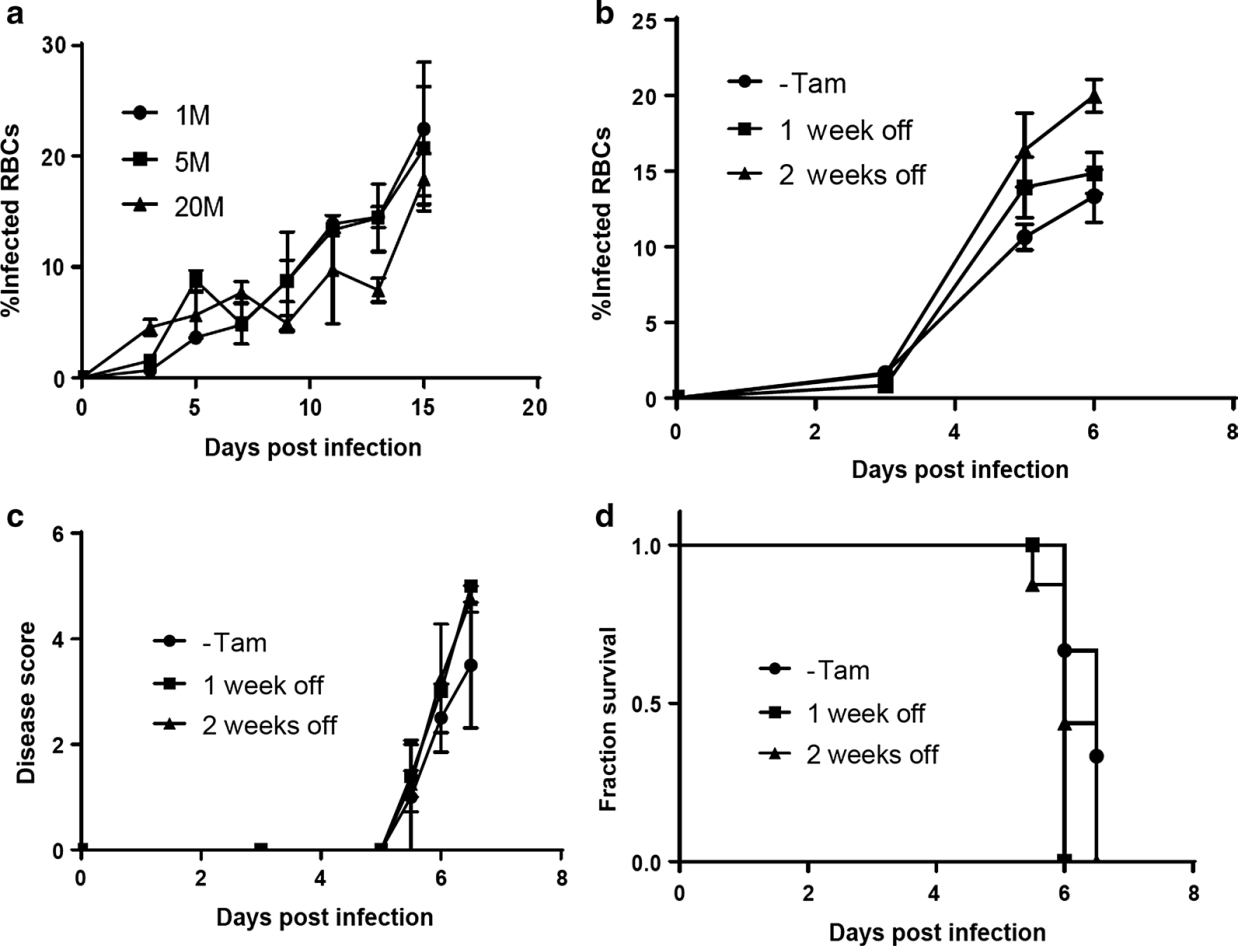
Alternative strategy to study cerebral malaria in mouse models that require tamoxifen. CB57/B6 mice were treated with tamoxifen 40 mg/kg per day one week prior infection with 1 million (circles), 5 millions (squares) or 20 millions (triangles) *P. berghei* ANKA infected RBCs. During the course of the infection animals continued being treated with tamoxifen and parasitaemia levels were monitored showing no differences between groups (**a**). As an alternative approach to study cerebral malaria in mouse models that require the use of tamoxifen, CB57/B6 mice were randomized into 3 groups: control group without tamoxifen (circles), treated with tamoxifen for 1 week and then rested, without any further tamoxifen treatment, for 1 week (squares) or 2 weeks (triangles) before infection with 1 million *P. berghei* infected RBCs. All groups developed comparable levels of parasitaemia (**b**), developed neurological signs of cerebral malaria (**c**) and did not differ in survival (**d**)

Since the induction of cerebral malaria in mice is dependent on the amount of inoculum used for infection [10], we tested whether the inhibition of cerebral malarial development by tamoxifen could be overcome by increasing the inoculum dose. Three groups of mice were subjected to tamoxifen treatment for 1 week prior to infection with 1, 5 or 20 million *P. berghei* infected RBCs. Parasite growth and signs of cerebral malaria were monitored thereafter, with the mice subjected to continuous tamoxifen treatment. Results show that all the groups had similar parasite levels throughout the experiment (Fig. 4a) and none of the mice in any of the treated groups developed signs of cerebral malaria.

The next step was to investigated whether an alternative approach where tamoxifen would be used prior to infection to induce the required Cre activity, followed by a period without treatment to allow the drug to be eliminated from the mice, would result in normal growth of the parasite and the development of cerebral malaria. To test this hypothesis, mice were treated with tamoxifen for 1 week and then rested, without any further tamoxifen treatment, for 1 or 2 weeks. Thereafter, mice were injected with 10^6^ *P. berghei* infected RBCs and carefully monitored for parasitaemia, disease signs and survival. These results show that resting for either 1 or 2 weeks post tamoxifen treatment enables parasite growth to levels similar to control untreated mice (Fig. 4b). Signs of cerebral malaria also increased similarly in all groups (Fig. 4c). Signs started to appear in all groups at day 5.5 post infection and the severity of the disease was comparable at all times. Survival was also similar between the groups (Fig. 4d), with all mice dying by day 6.5 post infection. Hence, these data demonstrate that tamoxifen directly affects the survival of *P. berghei,* however, pretreatment with tamoxifen does not significantly influence parasitaemia and the course of cerebral malaria when administered at least 1 week before infection.

## Discussion

Regardless of the constant efforts to reduce the burden of the disease, malaria was responsible for more than 260,000 deaths in children under 5 years in 2017 alone [4]. Emergence of new resistant strains of *Plasmodium* parasites to classical anti-malarial drugs, but in particular to artemisinin-based combination therapy [11, 12], are jeopardizing the international efforts to combat malaria and increase the need for the development of new therapies against this infection. Here we report the anti-malarial effects of tamoxifen, a drug that has been used worldwide for the treatment of breast cancer for over 30 years. Our observation that tamoxifen has potent activity against *P. falciparum* in vitro and induces a significant decrease of *P. berghei* growth in mice, positions this drug as a possible candidate for development as an anti-malarial. These data also suggest that patients taking tamoxifen for extended periods of time, as it is frequent when prescribed for prevention of recurrences or in genetically identified high-risk patients, may present increased protection against malaria.

Tamoxifen is also active against another protozoan parasite, *Leishmania*, with an IC50 of 11 µM in vitro [13], which is similar to these findings for *Plasmodium* (16 µM). Tamoxifen has been proposed as a partner in drug-combination therapies anti-*Leishmania*, since it increased the effectivity of classical agents against this parasite in mice with leishmaniasis [14, 15]. Additionally, it was shown that *Leishmania* is not prone to develop resistance to tamoxifen, which is an important feature for diseases such as malaria and leishmaniasis where drug resistance is frequently found in the field [16].

Of all malaria cases, only 1% of the patients develop severe malaria, a life-threatening disease [17]. The most common complications of severe malaria are cerebral malaria, acute respiratory distress, severe anaemia and acute kidney injury [18]. Patients of severe malaria can rapidly deteriorate, and mortality rate can exceed 50% even when anti-malarial treatment is provided [19]. Advancing the understanding of severe malaria pathogenesis is essential to develop new adjunctive therapies to treat these deadly complications of malaria. In that sense, rodent malaria models of human severe malaria are key to basic discovery, drug testing and pathogenesis research [20]. Rodent malaria models have raised some criticisms in the scientific community due to their limitations in reproducing the human disease; however, they have provided critical insights into the pathophysiological mechanisms involved in severe malaria [21]. This study shows that the susceptibility of *Plasmodium* to tamoxifen would interfere with the use of the Cre/loxP site-specific recombination system for the study of malaria since it interferes with the levels of infection and prevents the development of experimental cerebral malaria. Here is presented an alternative protocol that can overcome this problem by allowing the mice to clear tamoxifen levels during a week after treatment. Since only 0.3% of tamoxifen administered orally reaches the circulation, and blood tamoxifen concentration decreases by ~ 90% at 7 days post administration in mice [22], it is expected that the levels after 1 week would be negligible. It is important to note that this protocol may not be adequate when Cre recombination needs to occurs in tissues with high turnover rates, such as epithelial cells, since recombinant cells would reach the end of their life span and be replaced before the end of the week resting period.

*Plasmodium* blood-stage infection is confined to the circulation and associated immune organs, which are mostly constituted by cell types with low turnover rates, such as endothelial [23], and leukocytes [24], therefore, recombinant cells are expected to remain functional in the tissues for sufficient amount of time to complete a *Plasmodium* infection.

## Conclusion

Tamoxifen and its active metabolite, 4-hydroxytamoxifen, have antiplasmodial activity in vitro and in vivo. Since tamoxifen is an inexpensive, well-tolerated drug with an adequate safety profile, development of this drug for use in anti-malarial therapies may be warranted.

However, this anti-*Plasmodium* activity may also jeopardize the use of the conditional mutagenic Cre/LoxP system in the setting of rodent experimental models of malaria.

As an alternative strategy, we propose administrating tamoxifen for 1 week and keeping mice free of tamoxifen for another week prior to infection and after. With this approach, parasitaemia and cerebral malaria signs can be restored to control levels. This alternative strategy would be appropriate to investigate specific gene function in tissues with a low turnover, such as endothelium or glia.

## Data Availability

The datasets used and/or analysed during the current study are available from the corresponding author on reasonable request.

## Abbreviation

RBCs: red blood cells (erythrocytes)
